# Effect of Different Welding Modes on Morphology and Property of SS316L Stainless Steel Deposition by Robotic Metal-Inert Gas Welding

**DOI:** 10.3390/ma17184479

**Published:** 2024-09-12

**Authors:** Wei Wu, Chunjie Wen, Jisheng He, Yanfeng Li, Wei Xu, Ping Yao, Xiangkun Zeng

**Affiliations:** 1 School of Automobile and Transportation Engineering, Guangdong Polytechnic Normal University, Guangzhou 510450, China; wuwei_5v@126.com (W.W.); wenchunjie666@163.com (C.W.); ai81897335@gmail.com (J.H.); lyflwl88@163.com (Y.L.); xuy_wei@126.com (W.X.); 2 School of Electrical and Mechanical, Guangdong Polytechnic Normal University, Guangzhou 510635, China

**Keywords:** MIG welding, arc mode, morphology, property

## Abstract

The widespread adoption of arc additive manufacturing techniques across various industries has advanced the field of SS316L stainless steel manufacturing. It is crucial to acknowledge that different welding modes exert distinct influences on the forming and mechanical performance. This study analyzed the thermal input associated with four specific welding modes in LORCH MIG welding, clarifying the transition dynamics of molten droplets through waveform analysis and examining the resultant effects on microstructure and performance characteristics. The Pulse, Speed-Pulse-XT, and Twin-Pulse modes were found to induce spatter during the manufacturing process, consequently reducing molding efficiency in comparison to the SA-XT mode. Notably, the Twin-Pulse mode, characterized by double-pulse agitation, generated fish scale patterns along the lateral surfaces of the fabricated parts, promoting anisotropic grain growth. This microstructural refinement, compared to single-pulse samples with equivalent thermal input, resulted in enhanced mechanical properties. Nevertheless, the horizontal tensile strength of the three pulse modes was lower than the industrial standard for SA-XT mode and forging. In contrast, the SA-XT mode with an average hardness of 168.1 ± 6.9 HV and a tensile strength of 443.58 ± 5.7 MPa. Therefore, while three pulse modes offer certain microstructural advantages, the SA-XT mode demonstrates superior overall performance.

## 1. Introduction

Wire arc additive manufacturing (WAAM) is a technology that utilizes the arc generated by welding as an energy source. Based on the principle of layered deposition, it employs a wire feed system to transport materials layer by layer, enabling a rapid accumulation and forming process [1]. This method is known for its efficiency, speed, and cost-effectiveness, making it ideal for small batch and single production runs [2]. WAAM holds significant long-term development prospects in aerospace, marine transportation, medical, military, and other fields [3,4,5].

Deposition rates of WAAM based on gas metal arc welding (GMAW) are 2 to 3 times faster than gas tungsten arc welding-based or plasma arc welding-based WAAM, it has a wider range of materials, a higher deposition efficiency, and low-cost fabrication and repair of large high-density components [2,6]. Numerous studies have shown that GMAW could deposit different types of steel and improve properties. For example, Zhao et al. [7] studied the characteristics of arc manufacturing of stainless steel, analyzing the melting drop transition forming combined with specific parameters and providing the process parameter basis for arc additive manufacturing of stainless steel. Then, Gordon et al. [8] made 304 stainless steel parts with better GMAW fatigue resistance than as-cast parts. Furthermore, Pramod et al. [9] prepared 347 stainless steel (SS347) plates and found that, compared with the deformed alloy SS347, the tensile strength of the arc additive component was improved. The above research showed that additive manufacturing based on GMAW can be well applied.

However, different welding modes have different droplet transfer modes, welding currents, voltage waveforms and wire feeding speeds in GMAW, and produce different penetration depths and properties of weld [10,11]. Finally, they have varying influences on the forming and performance of additive manufacturing. Research on various arc modes has been categorized into three primary areas. Several studies have focused on the welding modes utilized in MIG welding systems. For instance, Wang et al. [12] conducted WAAM experiments using 316L stainless steel under both SpeedPulse and SpeedArc modes. Their research explored the mechanisms and effects of these arc modes on manufacturing process stability, structural integrity, microstructure evolution, and mechanical properties. The findings revealed that under identical deposition rates and scanning speeds, SpeedArc WAAM exhibited a reduced heat input and increased cooling rate compared to SpeedPulse WAAM. Consequently, this led to a finer solidification microstructure and enhanced tensile strength in the resultant material. Additionally, Zhang et al. [13] conducted a comparative analysis of the formability in arc additive manufacturing across various current modes. Their study revealed that pulse and variable polarity current modes resulted in lower heat input compared to non-pulse current modes. As a result, the fabricated components exhibited finer grain structures, fewer defects, and superior mechanical properties. Moreover, this paper’s prior work [14] also investigated the performance of SpeedArc and SpeedCold modes using 0.8 mm diameter welding wire. 

Many researchers have concentrated on exploring the welding modes of Cold Metal Transfer (CMT) welding machines [15,16]. For instance, Ren et al. [15] conducted an arc additive manufacturing study on thin-walled 316 stainless steel specimens using three distinct CMT modes: CMT, Pulse, and CMT + Pulse. Their comparative analysis of the microstructures and mechanical properties demonstrated that the CMT + Pulse mode offered the best forming performance. In a related study, Rodriguez et al. [16] examined the additive manufacturing of 316L stainless steel using CMT methods under continuous and pulse current modes. They compared single-layer weld formation, molding efficiency, and surface ripple effects between these modes. Their findings revealed that CMT + Pulse welding achieved a deposition rate of 3.7 kg/h while also satisfying the tensile performance criteria. 

Several scholars have conducted extensive studies on the various welding modes of CMT welding machines. For example, Hou et al. [17] utilized CMT+Pulse and CMT welding modes to perform arc additive manufacturing of 304 austenitic stainless steel, analyzing the resulting changes in mechanical properties. Additionally, Cong et al. [11] applied four different welding modes (CMT, CMT + Pulse, CMT advanced, and CMT + Pulse advanced) to fabricate Al-Cu alloys. Their findings indicated that the CMT + Pulse advanced mode was the most suitable for the WAAM process of this aluminum alloy, a conclusion also supported by Liu et al. [18]. In contrast, Chen et al. [19] investigated the manufacturing of WE43-Mg using these modes. Their results demonstrated that components deposited using the CMT mode exhibited superior mechanical properties, whereas the CMT + Pulse mode resulted in lower porosity.

Additionally, some researchers have conducted comparative studies on MIG and CMT welding in additive manufacturing. Prado-Cerqueira et al. [20] investigated the effects of MIG, CMT, variable polar CMT, and continuous CMT modes on the additive manufacturing of ER70S-6 low-carbon steel. Their study focused on analyzing the molding morphology, microstructure, and mechanical performance. The results indicated that continuous CMT additive manufacturing could effectively minimize the impact of the arc on both ends of the deposition, leading to superior molding quality. Moreover, they found that the hardness and strength of samples produced through MIG additive manufacturing were higher than those achieved using the three CMT modes.

Despite the extensive research on arc additive manufacturing using GMAW steel material, the LORCH S3 Robot MIG XT welding power supply offers a variety of welding modes. These modes possess varying thermal inputs and droplet transition forms, which in turn have diverse effects on additive manufacturing forming and performance. However, there is a lack of reports on the additive manufacturing of various welding modes, process parameters, and their influence on microstructure and performance. Therefore, this paper aims to explore the optimization of the arc additive manufacturing process, elucidate the different modes of MIG welding, and analyze the changes in structure and performance in arc additive manufacturing. This research will enhance the development of efficient arc additive manufacturing technology, broadening its applications in aviation, medicine, automotive, marine, and other industries.

## 2. Materials and Methods

In the WAAM process, heat input significantly influences the forming, microstructure, and mechanical properties of the produced components. SS316L stainless steel welding wire with 1.2 mm diameter and a 250 mm × 100 mm × 5 mm base plate were selected for the test. The LORCH S3 Robot MIG XT welding power supply (LORCH, Auenwald, Germany), provides a range of welding modes, each characterized by distinct welding parameters. Specifically, #SA-XT, #P, #TP, and #SP-XT correspond to the deposition of 1.2 mm diameter wire across 30 layers using Speed Arc-XT, single-pulse welding, twin-pulse welding, and Speed Pulse-XT, respectively. Then, the characteristics and advantages of different arc modes were shown in Table 1.

**Figure 1:**
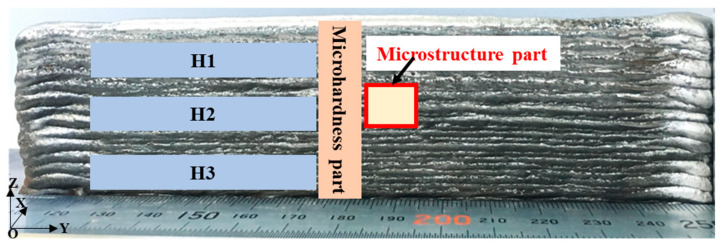
Test sample cutting position.

With a reciprocating-type method [21], a standardized interlayer cooling time of 30 s is maintained. The welding machine adopts a unified adjustment of parameters, that is, setting the same wire feeding speed, and then matching the corresponding welding voltage and current. Table 2 details the deposition parameters for these welding modes, including consistent wire feeding and scanning speeds to ensure uniform deposition rates. The specimen’s dimensions are 16 cm in length and under 50 mm in height, with the tensile specimen measuring 40 mm total length and featuring a gauge distance of 12 × 3 × 1.5 mm. Figure 1 illustrates the horizontal cutting method employed for tensile specimen preparation, focusing solely on the middle section for microscopic metallographic analysis, following the procedures outlined in Reference [14], H1, H2, and H3 were zones for tensile tests, and corresponded to the upper, middle, and lower sections. In accordance with the GB/T 228-2002 standard [22], tensile tests were conducted parallel to the welding scanning direction at a speed of 2 mm/min at room temperature. According to the GB/T 4340.4-2022 standard [23], the microhardness value along the deposition direction of the cross-section at 200 g load was measured with a microhardness meter (MVS-1000D1) (SHIMADZU Ltd., Kyoto, Japan) and maintained for 15 s.

**Table 1:** Characteristics and advantages of different arc modes.

Arc Modes	Metal Transfer Modes
SA-XT (Short-Circuiting Transfer) [24]	Characteristics and Advantages in AM
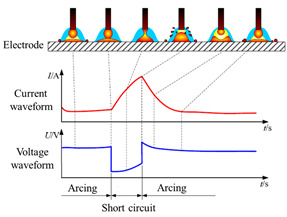	In welding arcs, a short circuit occurs when molten droplets form a short circuit between the electrodes. When the welding current is sufficiently high, the molten droplets come into contact with the arc between the welding wire and the workpiece, causing the arc to short-circuit and creating a brief high-temperature state. The short-circuit transition mode exhibits lower thermal input, which helps to reduce thermal deformation and residual stress in the components. The stability of the metal droplets in the molten pool is improved, leading to the formation of relatively uniform weld beads and enhancing the surface quality of the components. This mode demonstrates good adaptability to variations in materials and process parameters, making it suitable for additive manufacturing of various metallic materials.
P (Single Pulse Transfer) [25]	Characteristics and Advantages in AM
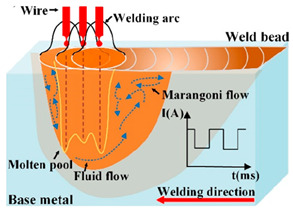	During the welding process, only a single pulse releases heat in each welding cycle. This mode controls the formation and transition of molten droplets through a single current pulse. The droplet transition during a single pulse process results in periodic fluctuations in the weld pool temperature, affecting the control of the weld pool and generating some degree of spatter. By controlling the frequency and amplitude of the current pulses, precise control of the metal droplets is achieved, which enhances the stability and accuracy of the deposition process, although some spattering is observed.
TP (Double Pulse Transfer) [26,27]	Characteristics and Advantages in AM
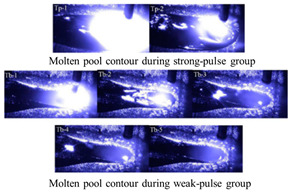 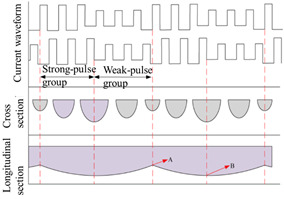	On the basis of single-pulse welding, low-frequency pulse modulation is introduced, resulting in two consecutive pulses in each welding cycle. This mode controls the formation and transition of molten droplets through the interaction of two pulses. The thermal input from the two pulses in the dual-pulse transition leads to periodic fluctuations in the weld pool between pulses. The dual-pulse transition provides more precise droplet separation and molten pool control, enabling higher deposition consistency and better mechanical properties. Improper low-frequency settings can lead to longitudinal shaping waves, and the dual-pulse transition may result in uneven thermal input, affecting the microstructure and performance of additive manufactured components.
SP-XT (Single Pulse Transfer)	Characteristics and Advantages in AM
The same as P	Similar to single-pulse transition, with lower current and higher voltage, a more refined droplet transition to the weld pool is achieved, which can improve the appearance and smoothness of the weld. The increase in voltage helps to extend the arc, but it can also lead to arc instability. Although the thermal input is lower, spattering occurs, which affects the forming process.

**Table 2:** Experimental parameters of different welding modes deposition (the thermal efficiency is 0.8).

Samples	Welding Modes	Layer	Current (A)	Wire Feed Speed (m/min)	Voltage (V)	Travelling Speed (cm/min)	Deposited Rate (kg/h)	Power (W)	Heat Input (J/mm)
#SA-XT	Speed Arc XT	30	171	4.8	19.1	60	0.924	3266	326.6
#P	Pulse		170		21.8			3706	370.6
#TP	Twin Pulse		170		21.8			3706	370.6
#SP-XT	Speed Pulse XT		137		22.6			3096	309.6

## 3. Results and Discussion

### 3.1. The Influence of Welding Modes on Forming Efficiency and Performance

#### 3.1.1. Stability and Arc Morphology Analysis of Deposition Process

According to the arc configurations specified in Table 3 of the LORCH welding machine operation manual [10], an analysis of the arc morphology for the process parameters utilized in this section was conducted. Table 3 reveals that #SA-XT, employing a 1.2 mm diameter wire, represents a short arc type. Conversely, #P, #TP, and #SP-XT are transition arcs characterized by lower currents and relatively lower stability. Despite these characteristics, the short arc method, noted for its deep penetration, narrow weld seam, and reduced spatter, facilitates a more stable droplet transition in arc additive manufacturing.

Data acquisition cards captured instantaneous voltage and current signals. Reference [28] indicates that the deposition process stabilized after reaching eight layers. Therefore, voltage and current waveforms were collected for the middle part of layer 8 across various welding modes, as detailed in Table 3. These waveforms varied among the four welding modes, with reduced fluctuations indicating a relatively stable deposition process [29,30].

**Figure 2:**
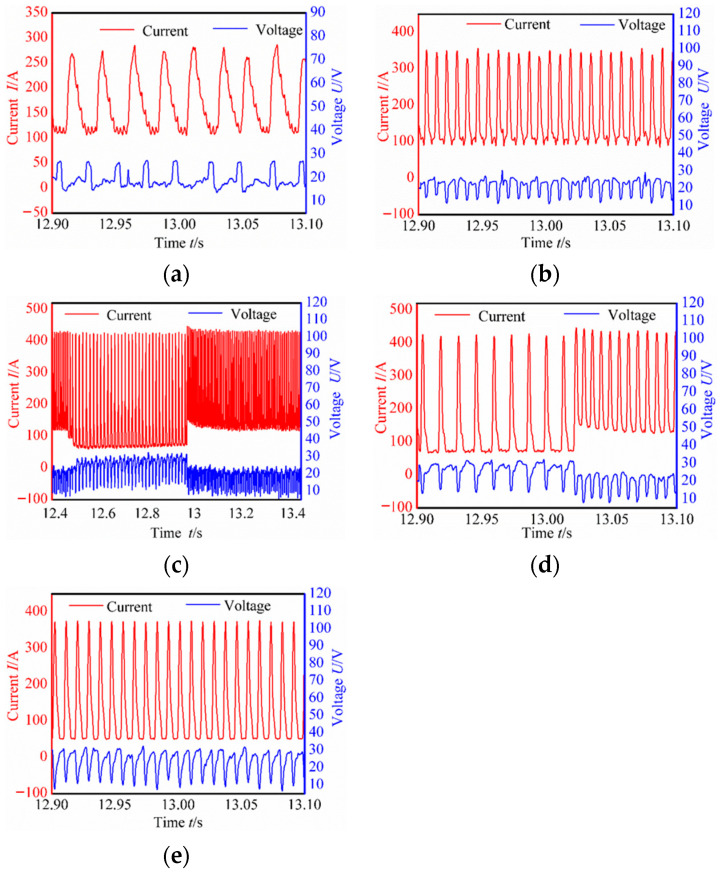
Waveform of different welding modes: (**a**) Speed Arc-XT mode; (**b**) Pulse mode; (**c**) Twin-Pulse mode; (**d**) enlarged picture of (**c**); (**e**) Speed Pulse-XT mode.

As shown in Figure 2, the Speed Arc-XT mode exhibited significantly reduced current at the same wire feed speed, enhancing current stability during the base value stage. And voltage increased with current. This mode not only achieved lower heat input deposition but also increased scanning speed with a voltage and current frequency of 45.7 Hz. The Pulse mode featured a base current overlaid with current pulses at a frequency of 125.7 Hz. In contrast, the Twin-Pulse mode alternated between two distinct, continuously switching pulses. The double-pulse envelope was dense, with the weaker pulse averaging 25% of the stronger pulse’s current, exhibiting a relatively stable waveform. The Speed Pulse-XT mode utilized an improved I-I-I controlled, non-short-circuit pulse welding process with a consistent operational frequency of 110 Hz, combining pulsed arc and droplet transition characteristics to achieve higher speed and deeper penetration welding. Its cleaner waveform compared to the pulse modes indicated a more stable deposition process [12].

**Figure 3:**
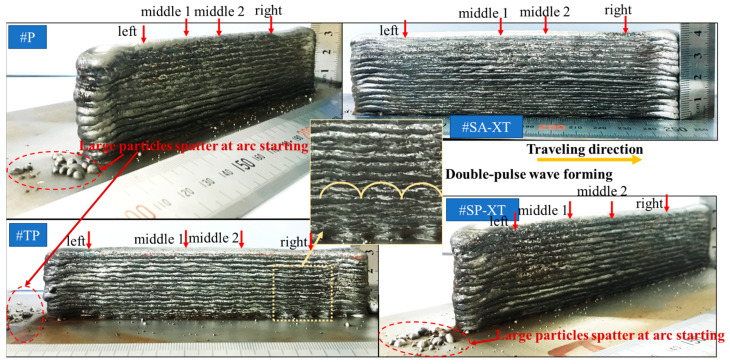
Profile morphology of different welding modes.

As illustrated in Figure 3, during the #SA-XT deposition process, the absence of short circuits was attributed to an arc voltage exceeding 15 V, ensuring continuous arc stability even at currents exceeding 40 A. Both current and voltage exhibited excellent repeatability across each cycle, indicating stable droplet transition and additive manufacturing processes. Conversely, #P and #TP exhibited spattering at the lower layers and both deposition ends, while maintaining stable upper-layer waveforms. The #TP was set to a low frequency of 1 Hz, with strong and weak pulse frequencies corresponding to current values of 128.9 Hz and 69.1 Hz, respectively. Audible double-pulse transition sounds were evident during additive manufacturing, accompanied by distinctive fish scale streaks on the molded side.

Contrasting with #P, the #SP-XT waveform demonstrated enhanced stability, with a reduced base current of 50 A exhibiting smooth operation and minimal splattering throughout the deposition process. Overall, each welding mode represented a relatively stable additive manufacturing process, with reinforced welding modes exhibiting lower heat input and more stable deposition characteristics.

#### 3.1.2. Macroscopic Morphology Analysis of Samples

The wire feed speed (deposition rate) of the 1.2 mm diameter welding wire remained consistent across different welding modes. However, Figure 3 highlights distinct forming morphologies in welding wire deposition across these modes. Notably, #SA-XT exhibited the highest deposition rate, whereas #TP displayed the lowest. The reinforced mode operated with lower current and higher voltage compared to non-reinforced modes, resulting in reduced total power and a more stable deposition process.

Significantly, large splatter particles were visible at the arcing ends of the three pulse modes which were highlighted inside the red dashed circle in Figure 3, contrasting sharply with the absence of such particles in the #SA-XT mode. In the #TP double-pulse mode, longitudinal fish scale stripes were prominent on the component’s side, concentrated within the yellow dashed line box in Figure 3. This effect was primarily attributed to the lower frequency of the double-pulse, emphasizing the formation of pronounced fish scales.

Different energies have an effect on bead geometry [31]. Due to the larger current applied to the 1.2 mm welding wire, resulting in rougher surface deposition, cross sections were cut and summarized in Table 3 according to the references [28,32,33]. The end sections appeared significantly shorter, wider, and rougher compared to the middle sections, primarily influenced by heat accumulation during the additive manufacturing process. Subsequent processing should adjust the width based on the middle section shape and height based on end section characteristics.

An image recognition forming evaluation system [22] tailored for additive manufacturing was developed utilizing the LabVIEW Vision 2015 software package. The system encompasses a comprehensive workflow including image calibration, pre-processing, binarization, feature extraction, and subsequent image analysis. Through this process, critical cross-sectional features of the fabricated samples were accurately extracted and identified. Key dimensional parameters such as the maximum height, maximum width, and the size of the inscribed rectangle for various sections were precisely measured, enabling a detailed assessment of the geometrical attributes of the additive-manufactured components. Comparative analysis showed consistent formation across all four sections of #SA-XT, exhibiting the smallest variance in average values and indicating minimal surface roughness. These findings, detailed in Table 4 and assessed using an image recognition forming evaluation system, revealed that #P > #TP > #SP-XT > #SA-XT in average effective width, while #SA-XT > #SP-XT > #P > #TP in height, which indicated the smallest heat accumulation due to lower heat input in #SA-XT and #SP-XT, resulting in taller layers and narrower widths. Moreover, pulse modes significantly perturbed droplet transitions in the molten pool compared to DC welding, contributing to more uniform formation in #SA-XT.

However, #TP’s double-pulse mechanism widened the sections and reduced heights by stirring the molten pool, while fish scale morphology increased side roughness and diminished effective width. 

Based on the height and width findings mentioned earlier, the forming parameters for each part are summarized in Table 5. For the #SA-XT sample, the maximum effective deposition rate and effective deposition rate per unit power were 0.82 ± 0.002 kg/h and 0.25 ± 0.001 kg·(h·kW)^−1^, respectively. Although the total deposition rates among the three pulse modes were identical, #P exhibited a marginally higher effective deposition rate than both #TP and #SP-XT. Notably, #SP-XT, operating at the lowest power, demonstrated the highest effective deposition rate per unit of power. Consequently, considering molding efficiency, Speed Arc-XT mode emerges as the optimal single-wire arc additive manufacturing mode for the LORCH welding machine utilizing a 1.2 mm SS316L stainless steel wire diameter.

### 3.2. Metallographic Structure Analysis of Samples

**Figure 4:**
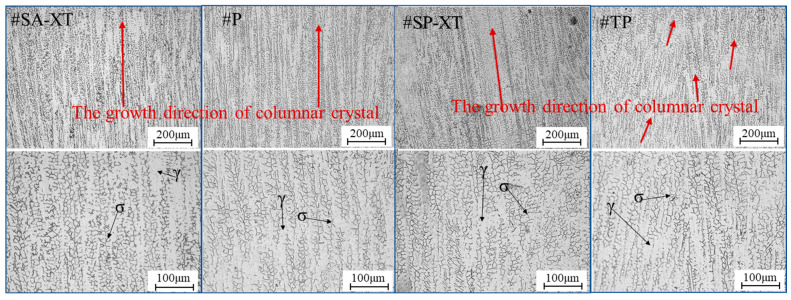
Microstructure of middle section of four modes samples with 1.2mm diameter.

Figure 4 illustrates microstructures in the central sections of samples from all four modes, featuring predominantly columnar crystals albeit with slightly varying growth orientations [34]. Austenite (γ) and ferritic (σ) phases [35,36] could be observed from the columnar dendritic structure in Figure 4. As a result of the thermal influence of the subsequent layer on the previous layer, part of the ferrite is dissolved in the austenite and the remaining ferrite exhibits a vermicular shape [37,38], yielding the final formation of cellular or reticular austenite. In #SA-XT, #P, and #SP-XT samples, columnar crystals primarily grew vertically upwards, whereas #TP displayed columnar crystals growing in diverse directions due to the stirring effect of double-pulse on the melt pool [39]. Notably, as highlighted by the red arrow, a significant presence of columnar crystals persisted due to the subtle stirring effect of low-frequency pulses.

The average secondary dendrite spacing measured by an image recognition system for #SA-XT, #P, #SP-XT, and #TP was 10.54 ± 0.51 μm, 13.35 ± 0.16 μm, 11.9 ± 0.63 μm, and 13.13 ± 0.06 μm, respectively. Notably, #SA-XT exhibited the smallest microstructure, while #P and #TP displayed relatively coarser microstructures, primarily influenced by variations in heat input and droplet transition modes [17].

### 3.3. Analysis of Mechanical Properties of Samples

#### 3.3.1. Hardness Analysis

**Figure 5:**
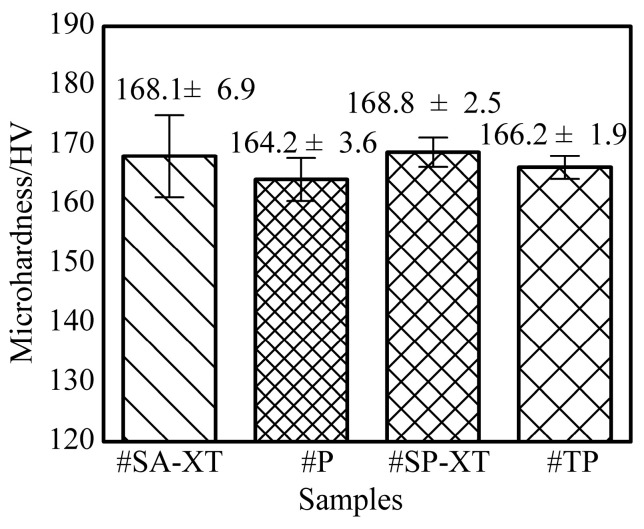
Average microhardness of samples.

Figure 5 illustrates the average hardness of #SP-XT, #SA-XT, #TP, and #P, highlighting the influence of different welding modes on additive manufacturing sample hardness. According to the Hall–Petch formula [40,41], grain size, influenced by heat input, primarily determines hardness. Smaller heat inputs result in finer grains, enhancing hardness by impeding metal deformation through increased grain boundaries.

High-speed solidification with low heat input contributed to higher hardness in #SP-XT (168.8 ± 2.5 HV) and #SA-XT (168.1 ± 6.9 HV). Despite similar heat inputs between #TP and #P, #TP (166.2 ± 1.9 HV) exhibited slightly higher hardness due to altered columnar crystal orientations and the dual-pulse stirring effect in the melt pool.

#### 3.3.2. Tensile Performance Analysis

From the horizontal tensile results presented in Table 6, it is evident that the strength distribution among the upper, middle, and lower sections of #SA-XT, #P, and #SP-XT samples conforms to the findings of Reference [10], indicating a pattern that the lower parts were greater than the middle parts, which were greater than the upper parts. However, this trend was not observed in #TP, possibly due to the stirring effect of double-pulse on the melt pool, influencing the vertical growth of columnar crystals.

**Figure 6:**
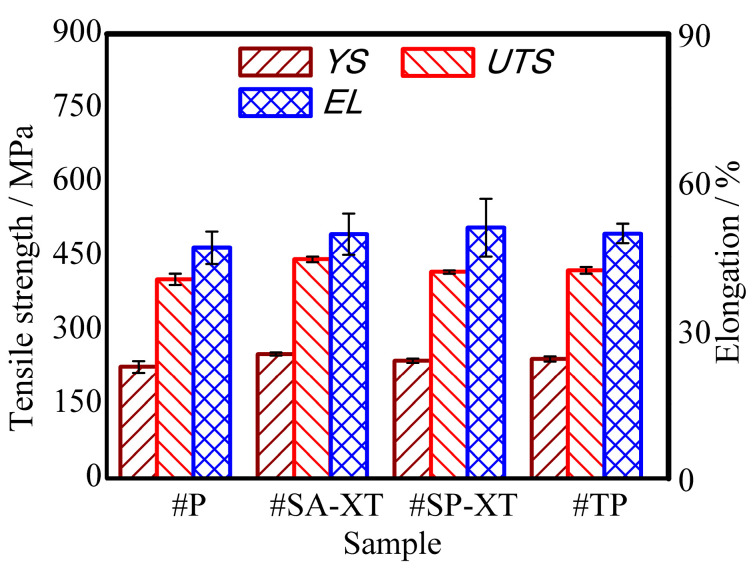
Column chart of tensile results.

Comparing the tensile strength across the four different modes in Figure 6, the order was #SA-XT > #TP > #SP-XT > #P, while the sequence of heat input was #P = #TP > #SA-XT > #SP-XT. This reaffirms that higher heat input tends to lower strength, but #TP, benefiting from its double-pulse stirring and grain refinement, demonstrated improved strength. The elongation values did not significantly differ among the four modes, all meeting industrial forging standards [42].

#SA-XT exhibited a maximum tensile strength of 443.58 ± 5.7 MPa and a yield strength of 252.01 ± 3.1 MPa, respectively, though slightly lower than the base metal’s strengths (383.13 MPa and 641.2 MPa) due to considerations for longitudinal stretch with high strength [43]. Conversely, the horizontal tensile strengths of the other three pulse modes did not meet the industrial forging standards. Hence, high-speed arc-reinforced welding proves more suitable for additive manufacturing in stainless steel MIG welding.

Based on the analysis presented, the strength and hardness of parts using a 1.2 mm diameter wire were found to be lower than those using a 0.8 mm diameter wire of 177.4 ± 4.8 HV and 563.97 ± 11.5 MPa, primarily due to approximately 3.3 times higher heat input [14]. This difference in diameter, welding speed, and cooling time results in a 1.28 times higher deposition rate for the 1.2 mm diameter wire, as derived from the deposition efficiency formula [24]. Consequently, while meeting the standards of the forging industry, employing the #SA-XT mode with a 1.2 mm diameter welding wire can further enhance forming efficiency, laying the groundwork for efficient additive manufacturing with double wires.

**Figure 7:**
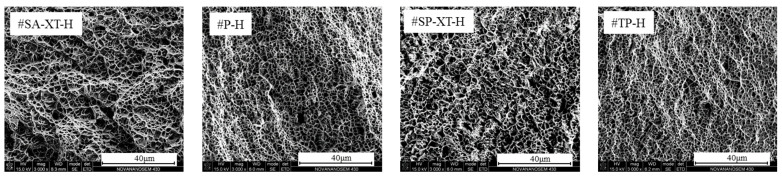
Microscopic fracture morphologies of tensile samples with different modes.

The microscopic morphology of horizontal tensile fractures depicted in Figure 7 revealed numerous equiaxed dimples and tear ridges. Notably, fractures in #SA-XT and #SP-XT exhibited relatively large dimples, indicative of superior plasticity [44]. In summary, regardless of the mode employed, the tensile fracture mechanism in MIG welding additive manufacturing of SS316L stainless steel predominantly exhibited ductile fracture characteristics.

## 4. Conclusions

This study employed an evaluation system to assess the impact of four welding modes in MIG welding on the formation and performance of deposition samples. The following conclusions were drawn:

(1) Compared to Speed Arc-XT welding, single-pulse welding, Speed Pulse-XT, and Twin-pulse modes all exhibited spattering during additive manufacturing, leading to lower molding efficiency. The effective deposition rate of Speed Arc XT welding was up to 0.82 kg·h^−1^. The Twin-pulse mode, characterized by double-pulse agitation, produced a fish scale stripe pattern on the side of the fabricated parts.
(2) Microstructure in Twin-pulse mode revealed columnar crystals growing in various directions, indicating a finer microstructure compared to Single-pulse samples with equivalent heat input, thereby enhancing the performance.
(3) Speed Pulse-XT welding exhibited slightly superior performance to single-pulse welding due to the lower heat input. However, the horizontal tensile strength of all three pulse modes fell short of both Speed Arc-XT and industry standards for forging. Speed Arc-XT demonstrated a hardness of 168.1 ± 6.9 HV and a tensile strength of 443.58 ± 5.7 MPa.

Considering the imperative of meeting industry forging standards, employing the Speed Arc-XT (SA-XT) welding mode with a 1.2 mm diameter wire proves advantageous for arc additive manufacturing, offering enhanced stability and efficiency in forming processes.

## Figures and Tables

**Table 3:** Welding modes with different processes of LORCH MIG welding machine [10].

Diameter of Welding Wire (mm)	Long Arc/Jet Arc		Transitional Arc		Short Arc	
	Current (A)	Voltage (V)	Current (A)	Voltage (V)	Current (A)	Voltage (V)
1.2	220~320	25~32	170~250	19~26	120~200	17~20

**Table 4:** Cross section of different positions in different mode depositions.

Samples	Left Height (mm)		Mid Width 1 (mm)		Mid Width 2 (mm)		Right Height (mm)		Average Height (mm)	Average Width (mm)
#SA-XT	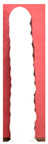	41.71	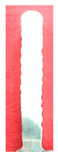	6.14	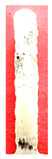	6.16	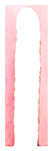	41.64	41.67 ± 0.03	6.15 ± 0.01
#P	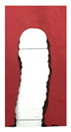	30.78	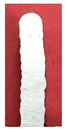	6.45	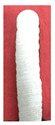	6.64	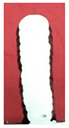	32.01	31.4 ± 0.61	6.55 ± 0.1
#TP	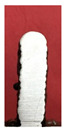	31.02	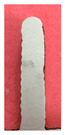	6.24	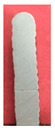	6.49	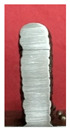	32.89	30.96 ± 0.07	6.37 ± 0.13
#SP-XT	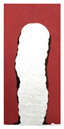	30.55	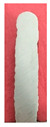	6.1	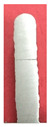	6.31	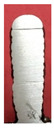	32.89	31.72 ± 1.17	6.21 ± 0.11

**Table 5:** Calculated values of depositions.

Samples	Total Volume $\left({\mathrm{cm}}^{3}\right)$	Effective Volume $\left({\mathrm{cm}}^{3}\right)$	Deposited Efficiency (%)	Average Power (kW)	Effective Deposition Rate (kg/h)	Effective Deposition Rate per Power (kg/(h·kW))	Roughness (mm)
#SA-XT	43.4	40.64 ± 0.1	88.26 ± 0.22	3.266	0.82 ± 0.002	0.25 ± 0.001	0.24 ± 0.06
#P		32.64 ± 1.14	75.21 ± 2.63	3.706	0.69 ± 0.024	0.19 ± 0.007	0.44 ± 0.07
#TP		31.3 ± 0.72	72.12 ± 1.65	3.706	0.67 ± 0.015	0.18 ± 0.004	0.53 ± 0.13
#SP-XT		31.26 ± 1.72	72.04 ± 3.96	3.096	0.67 ± 0.037	0.21 ± 0.012	0.48 ± 0.06

**Table 6:** Tensile results of deposition samples with different modes.

Tensile Properties Results	Samples	H1	H2	H3	Average of H
Tensile strength *UTS* (MPa)	#SA-XT	435.62 ± 5.2	446.32 ± 0.8	448.82 ± 3.1	443.58 ± 5.7
	#P	391.7 ± 6.1	398.42 ± 5.7	419.39 ± 2.3	403.17 ± 11.7
	#SP-XT	414.26 ± 2.7	417.97 ± 1.0	421.39 ± 0.2	417.87 ± 2.9
	#TP	428.6 ± 5.3	412.14 ± 0.5	422.62 ± 3.0	421.12 ± 6.8
Yield strength *YS* (MPa)	#SA-XT	249.58 ± 3.8	250.04 ± 3.6	256.42 ± 5.8	252.01 ± 3.1
	#P	213.67 ± 2.2	222.3 ± 0.6	242.11 ± 5.9	226.03 ± 11.9
	#SP-XT	244.62 ± 3.2	235.48 ± 6.1	234.88 ± 5.1	238.32 ± 4.4
	#TP	242.39 ± 6.3	235.57 ± 2.1	248.11 ± 4.5	242.02 ± 5.1
Elongation *EL* (%)	#SA-XT	48.3 ± 0.9	44.97 ± 3.2	55.22 ± 3.8	49.5 ± 4.2
	#P	42 ± 2.2	48.51 ± 0.72	49.72 ± 2.1	46.74 ± 3.3
	#SP-XT	43.23 ± 1.5	51.3 ± 5.5	57.86 ± 0.2	50.80 ± 5.9
	#TP	50.42 ± 3.7	46.86 ± 2.6	51.61 ± 2.9	49.63 ± 2.0

## Data Availability

The original contributions presented in the study are included in the article, further inquiries can be directed to the corresponding authors.
